# Respiratory Barrier as a Safeguard and Regulator of Defense Against Influenza A Virus and *Streptococcus pneumoniae*

**DOI:** 10.3389/fimmu.2020.00003

**Published:** 2020-02-04

**Authors:** Kim S. LeMessurier, Meenakshi Tiwary, Nicholas P. Morin, Amali E. Samarasinghe

**Affiliations:** ^1^Department of Pediatrics, College of Medicine, University of Tennessee Health Science Center, Memphis, TN, United States; ^2^Division of Pulmonology, Allergy-Immunology, and Sleep, College of Medicine, University of Tennessee Health Science Center, Memphis, TN, United States; ^3^Le Bonheur Children's Hospital, Children's Foundation Research Institute, Memphis, TN, United States; ^4^Division of Critical Care Medicine, College of Medicine, University of Tennessee Health Science Center, Memphis, TN, United States

**Keywords:** co-infection, lung mucosa, epithelial cells, barrier defense, respiratory tract

## Abstract

The primary function of the respiratory system of gas exchange renders it vulnerable to environmental pathogens that circulate in the air. Physical and cellular barriers of the respiratory tract mucosal surface utilize a variety of strategies to obstruct microbe entry. Physical barrier defenses including the surface fluid replete with antimicrobials, neutralizing immunoglobulins, mucus, and the epithelial cell layer with rapidly beating cilia form a near impenetrable wall that separates the external environment from the internal soft tissue of the host. Resident leukocytes, primarily of the innate immune branch, also maintain airway integrity by constant surveillance and the maintenance of homeostasis through the release of cytokines and growth factors. Unfortunately, pathogens such as influenza virus and *Streptococcus pneumoniae* require hosts for their replication and dissemination, and prey on the respiratory tract as an ideal environment causing severe damage to the host during their invasion. In this review, we outline the host-pathogen interactions during influenza and post-influenza bacterial pneumonia with a focus on inter- and intra-cellular crosstalk important in pulmonary immune responses.

## Introduction

**Figure 1:**
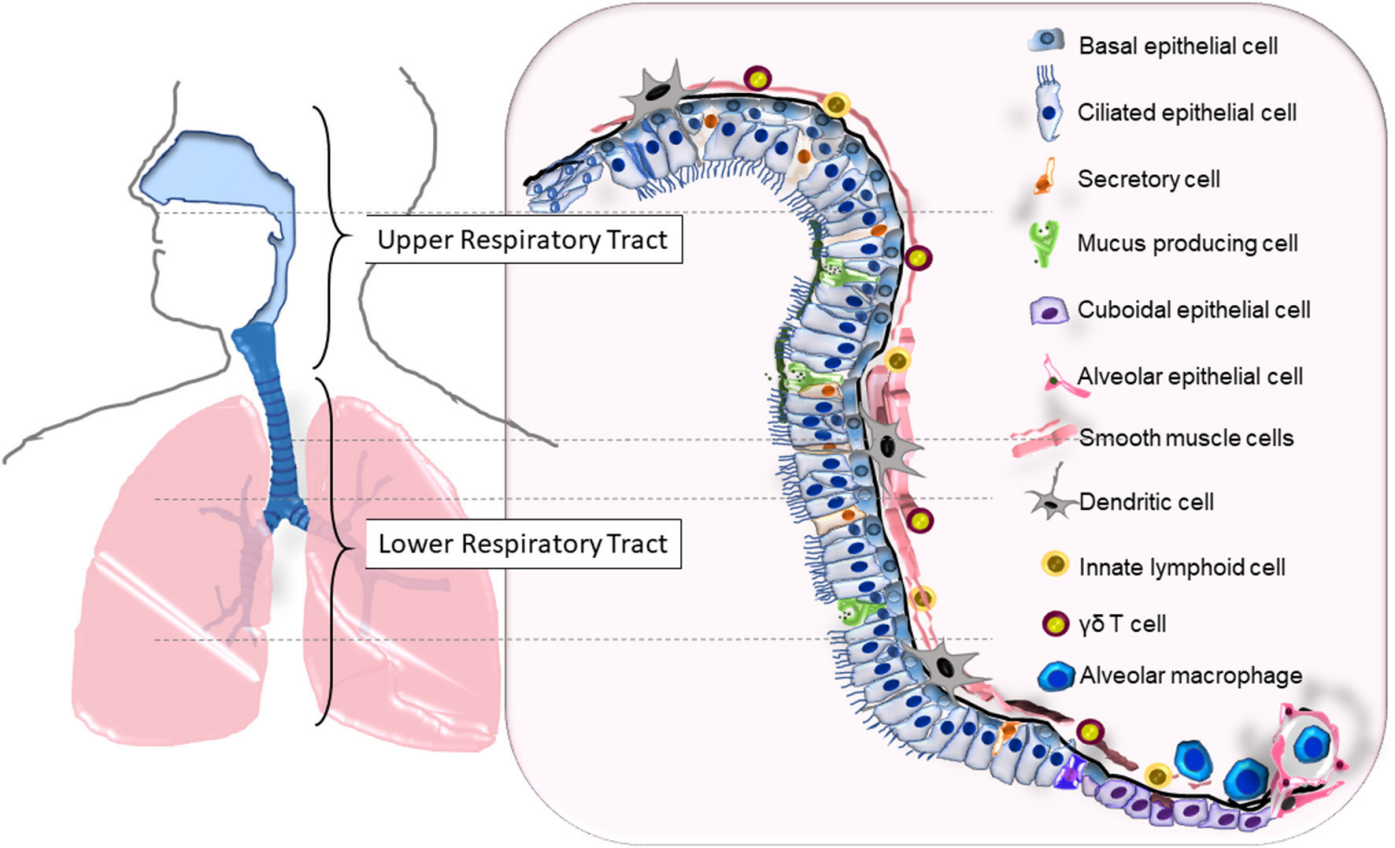
The cellular composition of the upper and lower respiratory tracts that serves as the primary barrier. Epithelial cells (ECs) that span the entire length of the respiratory tract (RT) are lined with basal cells that are attached to the basement membrane. Squamous ECs make up the beginning (nasal) and ends (alveoli) of the RT, ciliated and non-ciliated columnar epithelia makeup the upper RT and the large bronchi, while cuboidal epithelia line the small bronchi and bronchioles. Surface liquid that overlays the ECs consists of mucus secreted from mucus producing cells, airway liquids secreted from secretory cells, neutralizing immunoglobulins, and antimicrobials. Resident leukocytes such as dendritic cells, γδ T cells, and innate lymphoid cells line the mucosa while alveolar macrophages are found in the lower airways and alveoli. The bronchial smooth muscle cells underlying the RT from the basal end provide structural support and elasticity to the airways.

The respiratory system is divided into the upper (nasal passages, pharynx, larynx) and lower (trachea, bronchial tree, lungs) components with a cumulative mucosal surface area that exceeds 140 m^2^. The entire length of the system, roughly divided into the upper respiratory tract (URT) and the lower respiratory tract (LRT), contains a physical barrier made up of liquid and cell layers (Figure 1). The “one/united airway concept” was proposed to underscore the importance of considering changes that occur in the upper and lower airways concomitantly when investigating diseases that affect the respiratory tract like rhinitis and asthma (1). Approximately 2^23^ branches lined with epithelial cells make up the airways (2) within the soft lung tissue that handles ~10,000 L of inhaled air each day, placing this epithelial surface in contact with various noxious and innocuous material including environmentally disseminated viruses and bacteria.

As the primary point of contact, the epithelia of the respiratory system can be considered the regulatory point of immune responses at the respiratory mucosa. Made up of several types of epithelial cells, secretory cells, goblet cells and neuroendocrine cells, the mucosal barrier is multifunctional providing a physical barrier, secretory barrier, and immune defense (2, 3). Uniformity of upper and lower respiratory barrier components ensure multiple levels of filtration of air particles to safeguard the single-layer-thick alveolar spaces (Figure 1). When the secretory barrier consisting of mucus, antimicrobial proteins, neutralization antibodies, etc. is breached and epithelial cells come into contact with invading environmental pathogens, these cells become activated and begin communicating with resident leukocytes to participate in the inflammatory cascade and repair mechanisms that follow the invasion. In this review, we will discuss our current understanding of the barrier responses to two major respiratory pathogens, influenza A virus and *Streptococcus pneumoniae* in otherwise healthy hosts.

## Crosstalk Within the Mucosal Barrier During Influenza a Virus (IAV) Infection

Influenza is an infectious disease caused by influenza viruses belonging to the Orthomyxoviridae family. Of the four genera of influenza viruses, *influenza A* and *influenza B* are known to cause influenza in humans, with the former having a greater propensity to cause severe disease. Between 2010 and 2017, influenza illness in the United States affected 9–34 million persons and killed between 12,000–51,000 annually (4). As a segmented negative sense RNA virus, IAV is predisposed to genetic mutations and gene reassortment, the latter of which is supported by IAV's proclivity for zoonotic infections. Subtypes of IAV are based on the characteristics of surface expressed glycoproteins hemagglutinin (HA) and neuraminidase (NA) which also regulate viral binding and release during its life cycle within host cells. Although IAV has been shown to infect a variety of cell types (5), epithelial cells of both the upper and lower respiratory tracts are its primary target for replication (6, 7).

### Mechanisms of Inter-epithelial Crosstalk During IAV Infection

**Figure 2:**
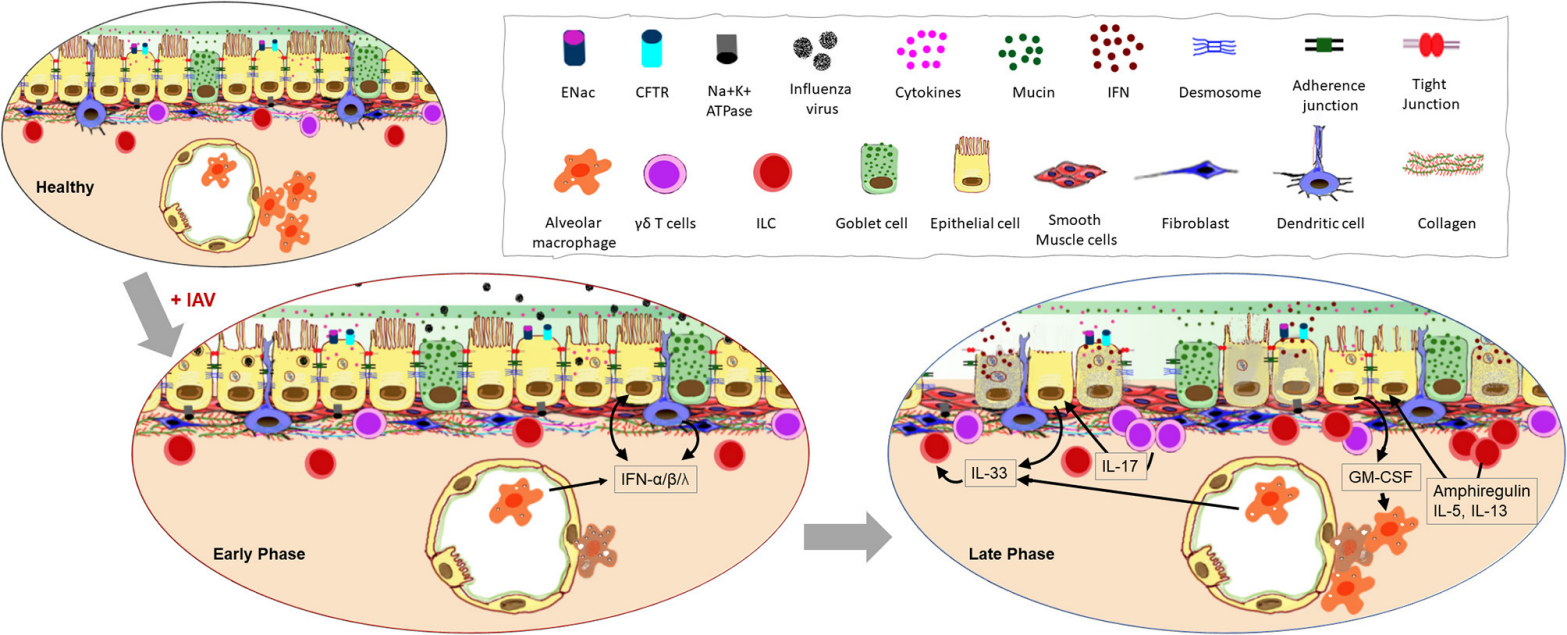
Impact of influenza A virus (IAV) infection on the respiratory barrier. Early infection of epithelial cells that express the sialic acid receptors causes damage to the physical barrier as junctional proteins become compromised during cell death. Increased cellular secretions and loss of cilia slow mucociliary clearance. Resident cells respond to the infection with type I and type III interferon (IFN) production and response. Continuation of these processes lead to the loss of epithelial cells thereby exposing the basement membrane. Morphological changes to the remaining epithelia further compromise the barrier response inducing leakiness in junctional proteins, inflammation, and aberrant repair processes.

Virus transmission is fundamental to IAV pathogenesis, and while its establishment in a new host is governed by HA molecules, environmental factors also play an important role in the distribution of mucosal secretions (large or small droplets and droplet nuclei) that contain infectious virions, as does human/animal behavior (8). Once IAV reaches the mucosa of the new host, it utilizes numerous strategies to overcome the hostile host environment for successful infection and pathogenesis. The airway epithelium consists of ciliated and non-ciliated cells overlaid by two layers of mucus (Figure 2); a bottom layer of less viscous periciliary liquid (PCL) which allows free ciliary movement and a top layer of gel-like mucus layer to which inhaled matter “sticks” (9). The mucus layer is also rich in various highly polymeric mucins (10), antimicrobial peptides (11), neutralizing antibodies (12), etc. that serve as a biochemical barrier to inhibit pathogen penetration (13). Most inhaled particles never gain access to the PCL as they bind to the gel layer and get brushed upward through the mucociliary escalator. Similarly, surfactant proteins that are abundant in lower airway secretions, bind to IAV and enhance viral clearance (14, 15). Virus attachment to the respiratory epithelia will be possible only for those infectious virions that bypass the upper gel barrier and gain access to the sol layer beneath. Viral HA protein facilitates its entry into the cell by binding to sialic acid receptors present on the apical side of epithelial cells. The linkage of sialic acid to the galactose could be either α-2,3 (recognized by avian viruses) or α-2,6 (recognized by human viruses) (16). Since sialic acid receptors are present as a heterogenous mix on epithelial cells in different species (17, 18), it is unclear how IAV selects its specificity and also why binding to sialic acids is usually limited to the URT epithelia (19) when these receptors are available throughout the airway epithelial barrier (17, 19, 20).

The physical manifestation of a barrier is afforded by three types of junctional proteins in the epithelia: tight junctions (TJ), adherens junctions (AJ), and desmosomes (Figure 2). Of these, the role of TJs is well-characterized during influenza virus pathogenesis. Three main transmembrane proteins [occludins, claudins, and junctional adhesion molecules (JAM)] are responsible for tightly sealing membranes of adjacent cells within the TJs. Peripheral membrane protein, zonula occludin (ZO), binds to these transmembrane proteins of the TJs to stabilize them in the cytoskeleton and mediate signaling (21–23). IAV infection disrupts the epithelial barrier by causing reduced expression of occludin, claudin-4, and JAM soon after infection (24). The non-structural protein 1 (NS1) of IAV plays a key role in virulence as the PDZ-binding motif (PBM) of NS1 binds to the PDZ domain present in TJ proteins (25) which then destabilizes junctional integrity through the rearrangement of ZO-1 and occludin (25).

During an active infection, the ability for host cells to communicate with one another is essential in order to warn surrounding cells of the threat and to initiate immune responses (Figure 2). Various strategies are employed by airway epithelia for this purpose including the release of interferons (IFNs) and other cytokines, antimicrobial peptides, nitric oxide (26), and the more recently described extracellular vesicles (27). The main viral countermeasure to these epithelial responses is the induction of epithelial cell death (28). Infection-induced production of type I IFN is known to trigger the expression of a variety of death-associated molecules in epithelia including Fas, TRAIL receptor, and caspases (29), causing epithelial cell death during the early phase of infection (30). The release of pro-inflammatory cytokines such as IL-1β initiated through inflammasome activation by IAV (31) can lead to pyroptosis (32). Virus-mediated epithelial cell death occurs early after infection with >50% death within 72 h (28), and since cell death increases permeability of the epithelial layer (33), productive infection of the respiratory epithelium is detrimental to barrier potency. Additionally, infected epithelia that present viral antigen-loaded MHC-I molecules are targeted by antigen-specific CD8^+^ T cells for destruction (34) which is a major mechanism of viral clearance in the lungs (35). Interestingly however, some ciliated and alveolar epithelial cells downregulate MHC-I and evade CD8^+^ T cell-mediated death to survive the IAV infection, showcasing a mechanism used by the immune system to reduce host pathology during influenza (36).

Epithelial cells of the lower respiratory tract terminate in the alveoli as squamous type I and type II pneumocytes (Figure 1). Since these cells are the primary site for gas exchange, they are bathed in a thin layer of fluid rich in surfactant proteins to reduce the surface tension with the adjoining capillary network of the lungs. One of the important functions of the alveolar epithelium is to remove fluid from the alveolar lumen with the help of ion channels such as amiloride-sensitive epithelial sodium channels (ENaCs), present on the apical surface of the pneumocytes (37, 38) and Na,K-ATPase present at the basolateral membranes (38, 39). Alveolar epithelia are also susceptible to IAV infection which leads to barrier destruction (40) thereby disrupting the intricate balance of ion transport and fluid maintenance causing edema, hypoxemia and pneumonia (38). In fact, IAV matrix protein 2 can inhibit ENaC to cause edema and respiratory insufficiency during influenza (41). Further evidence suggests that there is a cumulative downregulation of ENaC, CFTR, and Na,K-ATPase on epithelial cells during early stages of IAV infection (42). Interestingly, type I IFNs released by epithelia during the late phase of IAV infection, causes the upregulation of TRAIL on alveolar macrophages (AMs) which in turn causes epithelial cell Na,K-ATPase downregulation and edema (43). Alterations to the airway fluid dynamics affect all neighboring cells, infected or not, thereby influencing their functions. Similarly, epithelial cell-derived transforming growth factor (TGF)-β can be activated by viral NA (44) and can reduce the activity of Na,K-ATPase (45).

### Epithelial-Resident Leukocyte Crosstalk During Early IAV Infection

The respiratory mucosal barrier contains sentinel cells comprised of AMs, dendritic cells (DCs), γδ T-cells, and innate lymphoid cells (ILCs) which support the antiviral immune response at early and late phases of IAV infection as recently reviewed by us (46). While functional responses in each of these cells during influenza has been investigated, their interactions with the epithelium during an ongoing infection is not fully explored. Indirect communication between the epithelia and these resident leukocytes by means of cytokines may be of greater significance than direct interaction during IAV infection (Figure 2). Early release of cytokines from the infected epithelial cells regulate the tone of the immune response through activation of these resident cells.

Epithelial cells become aware of virus invasion mainly through three families of pattern recognition receptors; retinoic acid-inducible gene-like receptor (RLRs) (47), nucleotide-binding domain and leucine-rich-repeat-containing proteins (NLRs) (48) and toll-like receptors (TLR) (49), which, when stimulated, trigger the production of a variety of cytokines and chemokines including IFNs (Figure 2). While all three types of IFNs (type I, type II, and type III), are important in antiviral defense against IAV, type I and III are produced by the epithelia (50). The type I IFN receptor (IFNAR) is expressed on a variety of leukocytes in addition to the airway epithelial cells (AECs) allowing them to be responsive to IFNα and IFNβ (51, 52). Since the type III IFN receptor (IFNLR) is predominantly expressed on AECs, they are the most responsive to this cytokine (53). However, the discovery of the IFNLR on neutrophils and DCs suggests a more broad function for this cytokine during respiratory pathogen to protect the barrier response (54). Type II IFN is largely secreted by natural killer (NK) cells (55) and recruited CD8^+^ T cells (56) in response to IAV infection, and IFNγ signals the local macrophage populations that express the receptor IFNGR to promote phagocytosis, reactive bursts, and the production of proinflammatory cytokines (57).

Immediately following IAV infection, AMs contribute to the first wave of type I and type III IFNs, which are essential for the protection of the LRT from viral progression and dissemination (58, 59) and the virus needs to overcome this wave of IFNs if it is to establish a successful infection (60). Additional pro-inflammatory cytokines produced by AMs in response to IAV including TNFα, IFNγ, IL-1α, IL-1β, and IL-18 also contribute to enhanced viral clearance through the activation of antiviral defense mechanisms in surrounding immune and epithelial cells (61–64). However, a sudden and excessive production of cytokines (as are sometimes triggered by highly virulent strains of IAV), can cause alveolar hemorrhage, pulmonary edema, bronchopneumonia, and acute respiratory distress syndrome through damage to the mucosal epithelia (65–68).

The importance of AMs to all stages of respiratory immunity during influenza was highlighted by Ghoneim et al. wherein a virus-induced depletion of AMs in the lungs left the host vulnerable to invading opportunistic bacteria (69). Mice deficient in AMs are more susceptible to severe influenza due to increased infection of type I pneumocytes and diffuse alveolar damage (70). One critical growth factor for the differentiation, proliferation and activation of AMs is GM-CSF (71–73) which is largely produced by type II alveolar epithelial cells during influenza (74, 75) and mice deficient in GM-CSF (*Csf2*^−/−^), or its receptor (*Csf2rb*^−/−^) have increased morbidity and mortality during influenza similar to animals that are devoid of AMs (76) (Figure 2). Macrophages maintain environmental homeostasis through the removal of apoptotic cells and debris. As such, AMs are also important during the tissue repair phase that follows an active infection by IAV through the efferocytosis of dying epithelia and neutrophils (77). Epithelial cell proliferation and repair after influenza is promoted by AM products such as hepatocyte growth factor (78), TGF-α (79), and TGF-β (80).

Epithelial cell TLRs can guide the adaptive immune responses to IAV through molding the activation of DCs (81). Serving as a bridge between innate and adaptive immunity, DCs intersperse the epithelial barrier to sample inhaled air through dendrites. The majority of reports investigating the function of DCs during influenza have focused on their interaction with immune effectors that are recruited during the late phase of the immune response. Therefore, very little is known about the interaction of DCs with mucosal resident cells. Plasmacytoid DCs (pDCs) are known to produce high amounts of type I IFN during IAV infection through the TLR7/MyD88 pathway (82, 83). Human primary bronchial epithelial cells enhanced type I IFN production and the upregulation of IFN response genes in pDCs when co-cultured (84) showcasing crosstalk between the structural cells and local immune cells through cytokines. Similar crosstalk occurs between pDCs and AMs wherein pDCs control the number and cytokine profile of the AMs (85).

The airway epithelial barrier also contains a small percentage of γδ T cells that are considered to function in barrier defense. In murine models of IAV infection, γδ T cells increased during the late phase of disease (86), and produced immunoregulatory cytokines IL-2, IL-4, and IFN-γ (87). However, depletion of γδ T cells did not have any impact on viral clearance or IFN-γ production in a neonatal model of IAV infection in mice (88). Highly pathogenic H5N1 IAV can directly activate γδ T cells inducing the upregulation of CD69 expression and enhancing IFN-γ secretion (89). Similarly, γδ T cells produce IL-17A in response to IAV that triggers the release of IL-33 by AECs which in turn mediates ILC2s and Treg cells (88). These data indicate that γδ T cells are critical in maintenance of lung homeostasis and tissue repair during the viral clearance phase.

Additional protection and regulation to the mucosal barrier is provided by ILCs that are characterized by the absence of both T- and B-cell receptors. Like T-cells, ILCs have also been categorized according to cytokine production profile (90), of which ILC2 is the most investigated subset in the context of influenza. ILC2 is classically known to produce IL-5 and IL-13 in response to epithelial cytokines IL-25, IL-33, and TSLP (91). Infection of wild type as well as *Rag1*^−/−^ mice with IAV led to ILC accumulation in the lung (92) although there is no direct evidence that IAV-mediated ILC accumulation is dependent on AEC-derived cytokines. Furthermore, it has been reported that IAV infection induced AMs to produce IL-33 which promotes IL-13-dependent airway hyperreactivity (93). Its role in tissue homeostasis is implied in studies wherein ILC depletion was shown to impact lung function, epithelial integrity and tissue remodeling (92). The high amounts of type I and type II IFNs produced during the early phase of IAV infection have been shown to inhibit ILC2 function and proliferation (94). Conversely, IFN-γ deficiency leads to host protection through increased production of IL-5 and amphiregulin by ILC2 (94). Both NKT-cells and AMs have also been shown to produce IL-33 in response to IAV signaling ILCs to produce IL-5 (95), and increased levels of IL-5 during the viral clearance phase may help recruit eosinophils to the airway mucosal barrier (95) which can enhance cellular immune responses (96) and perhaps necessary for tissue repair (97).

## Opportunistic *Streptococcus pneumoniae* Infections

In some instances, virus-induced inflammation and dysregulated communication with the lung framework can leave the host vulnerable to secondary bacterial infections. This is exemplified by the increased susceptibility of an individual with IAV infection to the acquisition of *Streptococcus pneumoniae* (pneumococcus) (98, 99), resulting in a convergence that provokes far greater morbidity and mortality than infection with either pathogen alone (100, 101). The host remains susceptible to *S. pneumoniae* infection even after the virus itself has been cleared (102), suggesting that a compromised immune milieu and structural barrier contribute to increased bacterial pathogenesis. Although IAV can enhance *S. pneumoniae* pathogenesis directly, for instance by exposing cryptic binding sites through epithelial damage (103) or by liberating sialic acid and sialylated mucin that can be catabolized by *S. pneumoniae* (104), influenza virus can also modify interactions between the epithelium and inflammatory components, creating an environment that can be subverted by the pneumococcus.

### Impact of Influenza-Mediated Alterations to Epithelial Crosstalk on Pneumococcal Infection

Surface expressed TLRs on epithelial cells can sense *S. pneumoniae* by recognition of numerous bacterial components, including TLR2 agonists type 1 pilus, peptidoglycan, lipoteichoic acid and bacterial lipoproteins, and the TLR4 agonist pneumolysin (105–110). Although IAV is not directly recognized by either TLR2 or TLR4, the regulation and activation of TLRs during influenza has been shown to enhance susceptibility to secondary bacterial infection. Increased TLR2 signaling during IAV/*S. pneumoniae* co-infection results in heightened production of IL-1β, augmenting inflammation and morbidity (111). Additionally, IAV infection positively regulates TLR3 on pulmonary epithelial cells (112), which recognizes double-stranded RNA and impairs the clearance of *S. pneumoniae* from the lungs following activation by poly I:C (113). Stimulation of TLR3 also leads to early production of IFNβ by AECs (114), contributing to the type I IFN response elicited during influenza, which is a key factor in host susceptibility to secondary pneumococcal infection, as discussed later in this review.

A crucial initial step in pneumococcal pathogenesis is bacterial adherence to the respiratory epithelium. Initially, *S. pneumoniae* establishes itself in the host by colonizing the nasopharynx, which is considered a necessary precursor to pneumococcal disease (115). IAV-induced epithelial cell death may expose the basement membrane to which *S. pneumoniae* can bind to and use as a shortcut to the bloodstream (116, 117). Pneumococcus surface proteins including PavA and PavB, PfbA and PfbB, PepO and pilus subunit RgrA all have the ability to bind basement membrane components fibronectin, laminin, and collagen (118–122).

**Figure 3:**
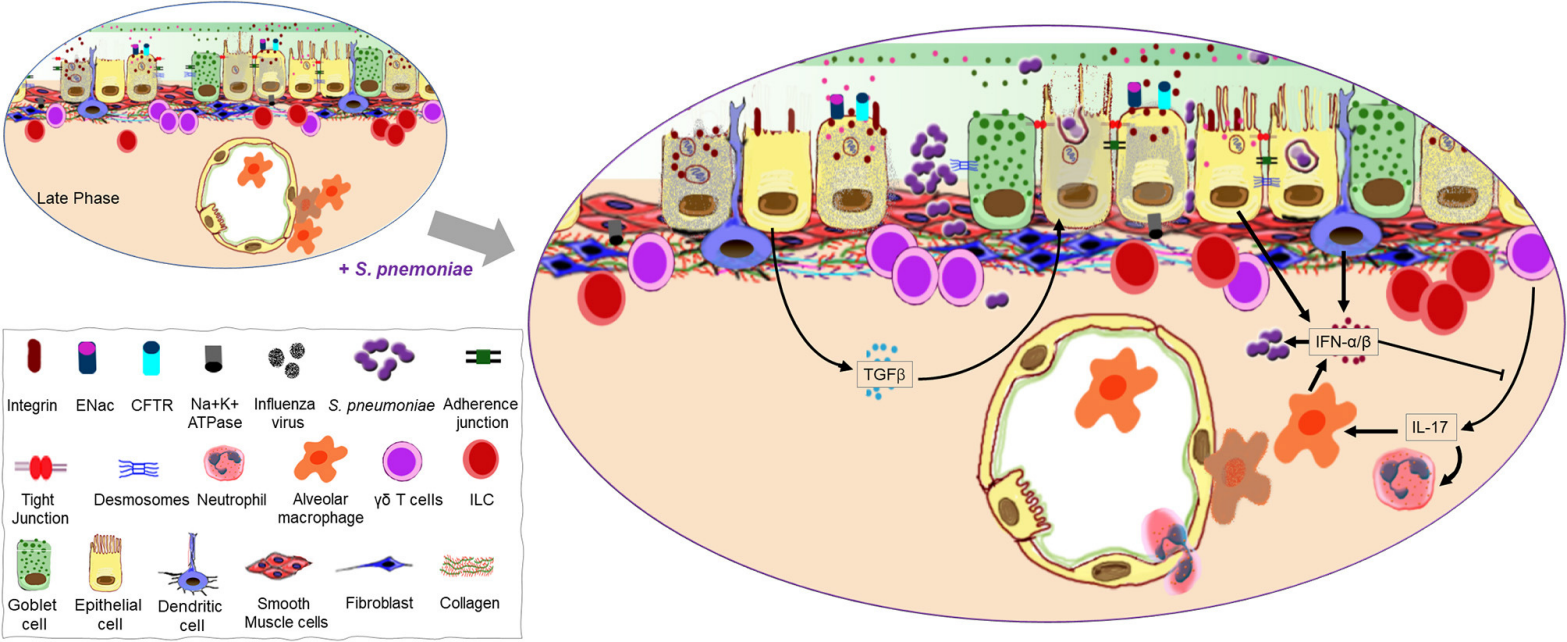
Continuation of the mucosal damage cascade permitting opportunistic infection. A host recovering from virus-induced damage to the lung mucosa is highly susceptible to *Streptococcus pneumoniae* infection possibly due to exposed binding partners on the host cells as well as an open barrier (gate) due to significant loss of epithelial cells. A second wave of type I IFNs may help promote bacterial colonization as it negates the positive influence interleukin (IL)-17 has on the recruitment of phagocytes. Transforming growth factor (TGF)-β produced during the late phase of influenza as a repair mechanism may also promote bacterial adherence to the mucosal surface.

From the URT, pneumococci can migrate to the lungs and establish symptomatic infections such as pneumonia and bacteremia (123). In a healthy individual, most wayward pneumococci in the airways are expelled by the mucociliary escalator before reaching the LRT (13). However, a recent IAV infection reduces the velocity of ciliary beating and causes death of ciliated tracheal cells, providing pneumococci an opportunity to bind to the epithelium observed as early as 2 h after challenge in mice (116, 124). In addition to increased access, IAV regulates binding receptors for *S. pneumoniae* on the epithelial surface (Figure 3). Numerous viruses, including IAV, can increase the prevalence of host platelet activating factor receptor (PAFr), which binds phosphorylcholine moieties in the pneumococcal cell wall (125–127). The activation of latent TGFβ by IAV NA present in the airways during influenza primes the epithelium for bacterial adherence by stimulating cells to upregulate bacterial receptors such as integrins (128). In the absence of TGFβ signaling, IAV-infected epithelial cells lose their increased vulnerability to pneumococcal colonization (129).

Although invasive disease is arguably not a favorable outcome for an extracellular respiratory bacterium like *S. pneumoniae* where optimal infection doesn't extend past the airways, prior influenza can promote its migration from the lungs to the bloodstream (130, 131). Under homeostatic conditions, however, the strict maintenance of TJs between cells in the epithelial and endothelial barriers prevents pneumococcal migration by physically restricting the movement of bacteria between cells and masking receptors. The disruption of TJs during influenza permits *S. pneumoniae* to migrate from the airways to the bloodstream. Pneumococci can also enter the blood from the airways by transmigrating through epithelial and endothelial cells (132–134). Following the binding of cell wall phosphorylcholine moieties to host PAFr, pneumococci can be internalized when the receptor is recycled (132, 135, 136) (Figure 3). Alternatively, invasion may be facilitated by the interaction of polymeric immunoglobulin receptor (pIgR) with pneumococcal CbpA and RrgA pillus subunit, the latter of which is involved in pIgR-mediated invasion of the brain microvascular endothelium, a mechanism that may also be applicable to nasopharyngeal epithelial cells (134, 137, 138). While levels of epithelial surface activation markers associated with bacterial defense EpCAM, IL-22Rα1, HLA-DR, CD40, CD54, and CD107a are not altered during pneumococcal colonization of the URT, bacterial uptake by pharyngeal epithelial cells is associated with strain-dependent changes to the transcriptome (139). While invasive strains like TIGR4 induce the upregulation of more genes compared to strains typically associated with carriage, the regulated pathways common to both colonizing and disease-causing *S. pneumoniae* strains are those associated with the innate immune response, such as NFκB and MAP kinase activation, toll receptor and cytokine signaling (139, 140) and correspond to hypersecretion of IL-6, and IL-8 (139). Furthermore, the most profound changes to the transcriptome following pneumococcal infection coincide with clearance of colonizing bacteria in an experimental human pneumococcal carriage model (139), suggesting that transmigration to the bloodstream may be an unintentional consequence during the innate resolution of pneumococcal carriage.

### Epithelial-Leukocyte Crosstalk During Pneumococcal Infection

During pneumococcal infection, IL-17 is produced by γδ T-cells (predominant source of IL-17 during pneumococcal pneumonia) and later by T_H_17 CD4^+^ T-cells. IL-17 and a T_H_17 response at the mucosal epithelium participate in pneumococcal clearance in the nasopharynx and lungs by recruiting monocytes and neutrophils, and offer protection against reinfection (141–144). However, the induction of type I IFN during influenza inhibits T_H_17 defense during secondary pneumococcal infection and suppresses the expression of IL-17 by pulmonary γδ T-cells, resulting in impaired recruitment of these phagocytes (129, 141, 145) (Figure 3). Furthermore, type I IFN also reduces the production of CCL2, leading to fewer recruited macrophages in the airways during a concurrent pneumococcal infection and increased colonization of the URT (146). Mice recovering from influenza are also unable to mount an effective KC and MIP-2 response following infection with *S. pneumoniae*, which stunts neutrophil recruitment (147). Macrophages and neutrophils are major components of the innate cell response against extracellular bacteria, controlling bacterial infection by phagocytosis, direct killing, and recruitment/activation of other inflammatory cells (148, 149). Early induction of type I IFN by AMs, DCs and AECs is of fundamental importance to antiviral immunity during influenza (150–154), but, can be detrimental during pneumococcal infection by disrupting the recruitment of cells that are important in controlling bacterial outgrowth (147). Accordingly, mice lacking IFNAR signaling have fewer bacteria in the lungs, lower levels of bacteremia and a better outcome following IAV-*S. pneumoniae* co-infection (147).

Mononuclear cells and neutrophils that are recruited to the airways during influenza contribute to damage of the respiratory epithelium. Recruited macrophages cause significant TRAIL-dependent apoptosis and leakage through the AECs (155). The increase in recruited macrophages is paralleled by a loss of AMs, hampering the host's ability to restrict a secondary pneumococcal infection which rapidly progresses to pneumonia (69). Neutrophil extracellular traps released in response to IAV are potentially damaging to the epithelium and are ineffective against secondary pneumococcal infection (156).

Pneumococci that enter the post-influenza RT not only are presented with an environment harboring reduced numbers of resident macrophages (69), but also encounter lymphocytes that are in a state of immunological exhaustion and unable to appropriately respond to the infection (157). Type I IFN produced by epithelial cells and others during IAV infection causes polyclonal activation of T- and B-cells which, despite the cells returning to a “baseline” state several days after infection, prevents activation by subsequent exposure to type I IFN. This state of exhaustion lasts for several days, during which the host is particularly vulnerable to secondary infections (157).

IAV infection is not solely good news for *S. pneumoniae*, with the host response to the viral infection also promoting protection against secondary bacterial infection in some instances. For example, while type I IFN disrupts cell recruitment during pneumococcal infection, its induction also restricts *S. pneumoniae* pathogenesis by up-regulating the expression of TJ proteins (ZO-1, claudin 4, claudin 5, claudin 18, and E-cadherin) and decreasing PAFr levels in epithelial and endothelial lung cells (158). Adenosine is present in the extracellular environment during stress and inflammation, and has been shown to be released by respiratory epithelial cells amongst others (159). During IAV infection of mice, ATP levels in the airways are elevated due to increased *de novo* synthesis and poor alveolar fluid clearance (160, 161), which can be sequentially hydrolyzed to generate adenosine (162, 163). The activation of A1-adenosine receptors by extracellular adenosine decreases expression of the PAFr on the lung epithelium (164) and promotes the recruitment of neutrophils, monocytes and lymphocytes during influenza (161), which contribute to protection against secondary infection with *S. pneumoniae* (164, 165).

IL-22 is produced during influenza by pulmonary NK cells (166) and RORγ^+^ αβ, and γδ T cells (167) and binds IL-22Rα1 on AECs and endothelial cells (168–170), an interaction that can be antagonized by its soluble form, IL-22BP (171, 172). Human endothelial cells respond to IL-22 by increasing production of CCL2 and CCL20 (169), which are chemoattractants for cells involved in the resolution of bacterial infection such as monocytes, dendritic cells, and lymphocytes. IL-22 is critical to epithelial repair following infection with A/PR/8/1934 (173), and in its absence, mice sustain significantly higher lung injury and loss of airway epithelial integrity during sublethal IAV infection followed by *S. pneumoniae* co-infection (167). Administration of exogenous IL-22 to mice with influenza causes the upregulation of genes encoding proteins involved in cell-cell adhesion such as *Cldn24* and *Pcdh15* (encoding claudin 24 and protocadherin 15, respectively) in the lungs, and reduces systemic dissemination of *S. pneumoniae* during secondary bacterial infection (174). Interestingly, although mice lacking the IL-22 decoy IL-22BP have significantly reduced bacterial outgrowth in the lungs during co-infection, dissemination is unaffected (175).

### Impact of IAV-Pneumococci Co-infection on Immune Defense at the Respiratory Barrier

The mucoepithelial barrier is one of the most important host respiratory defenses against encroaching bacterial pathogens. However, local damage and the inflammatory milieu occasioned during influenza can compromise the efficacy of the physical barrier and its interactions with other components of the inflammatory repertoire. It is interesting that many aspects of the post-influenza lung microenvironment known to exacerbate pneumococcal infection, are also targeted by *S. pneumoniae* in order to avoid immune clearance and establish infection. The pneumococcal cytotoxin, pneumolysin, disrupts TJs and reduces cilia organization and prevalence with negligible impact on ciliary beating (117, 176). In addition, *S. pneumoniae* causes cell damage and loss of planar epithelial architecture at the mucosal surface (117, 176). Pneumococci are able to evade neutrophils by expressing a polysaccharide capsule that also physically reduces deposition of complement and antibodies (177, 178), and by molecular mimicry wherein bacterial phosphorylcholine moieties bind PAFr, preventing PAF from initiating neutrophil phagocytosis and bactericidal activities (135, 179–181). In this respect, IAV is a perfect partner for *S. pneumoniae*, providing it with a compromised mucosal epithelial barrier that is permissive for it to establish infection, while at the same time dampening antibacterial host responses.

In reported *in vivo* models of co-infection, animals are commonly challenged with *S. pneumoniae* 3–7 days after IAV, corresponding to the most pronounced changes to morbidity and mortality (100, 182). However, influenza still predisposes mice to *S. pneumoniae* infection at later times of challenge, and clinically there are positive correlations between influenza and severe pneumococcal pneumonia with up to 4 weeks separating the two infectious agents, suggesting the IAV imparts long term effects in the host (183, 184). This is predictable, as IAV causes profound destruction of type II pneumocytes causing impaired regeneration after disease resolution, and also infects EpCam^high^CD24^low^integrin(α6β4)^high^CD200^+^ epithelial stem/progenitor cells thereby reducing renewal of cells at the respiratory barrier (185, 186). Influenza that precedes a pneumococcal infection may also affect the immune response during reinfection with *S. pneumoniae*. T_H_17 immunity promotes accelerated bacterial clearance in the URT following a secondary infection with *S. pneumoniae* (144). Considering that type I IFN inhibits T_H_17 activation (145) and thus the generation of memory cells, influenza may prevent T_H_17-mediated protection against subsequent infections with the same or heterologous pneumococcal serotypes (144, 187, 188).

## Targeting IAV and *S. pneumoniae* at the Mucosal Barrier

Clinical influenza disease commonly manifests as an uncomplicated upper respiratory infection with fever, malaise, headache, cough, and myalgias. Symptomatic treatment consists of over the counter anti-inflammatory and pain medications. The mainstay of current influenza antiviral medications are the NA inhibitors: oseltamivir, zanamivir, and peramivir. The sialic acid cleavage activity of NA is required for release of virions from infected epithelial cells and also facilitates migration through the epithelial mucin layer (189, 190). Benefit from NA inhibitors is primarily restricted to uncomplicated disease where treatment is instituted within the first 48 h of symptoms with a modest reduction in duration of illness (191, 192). A recently approved antiviral, baloxavir marboxil, acts as a selective inhibitor of influenza cap endonuclease (193). Similar to NA inhibitors, baloxavir marboxil has proven benefit in early treatment of uncomplicated influenza cases (193). Additionally, there was an observation of rapid development of resistance in outpatient trials raising concern for its long-term usage (194). Nitazoxanide is an antiprotozoal drug used to treat *Cryptosporidium* and *Giardia* infections. *In vitro* data demonstrate antiviral activity against influenza A and B strains (195, 196). It acts by inhibiting influenza HA trafficking through the epithelial endoplasmic reticulum and Golgi apparatus and preventing maturation by blocking HA terminal glycosylation (197). A phase 2b/3 trial of nitazoxanide in uncomplicated influenza was well-tolerated and showed reduced symptoms and viral loads (198). A randomized placebo-controlled phase III trial was completed in March 2019 and remains currently unpublished (196). If approved, this drug, through its primary targeting of the virus, will also affect the local immune responses to the virus initiated by the respiratory epithelial cells as detailed above.

Severe influenza can lead to respiratory failure and acute respiratory distress syndrome (ARDS) which has a mortality rate of 27–45% (199). Epithelial barrier disruption and pronounced pulmonary edema are hallmarks of ARDS and since there are no directed treatments that counteract these effects at present, and care remains predominantly supportive with mechanical ventilation, secretion clearance, and extracorporeal membrane oxygenation when necessary. As such, there is an evident need for additional influenza therapies, particularly for hospitalized patients with severe disease. As the primary site of infection, the respiratory epithelium represents an important area of focus for disease treatment. Fludase is a recombinant sialidase that cleaves the sialic acid receptor for IAV on AECs preventing viral entry into cells (200). Pre-clinical trials show broad *in vitro* influenza antiviral activity and protective effects in animal models (200, 201). In phase I and II trials, Fludase was well-tolerated and led to decreased viral load and shedding (202, 203). However, Fludase liberation of sialic acid raises interesting questions regarding *S. pneumoniae* co-infection as sialic acid has been shown to facilitate its colonization during IAV infection (104). *S. pneumoniae* infection of Fludase-treated mice with influenza did not alter bacterial colonization or mortality (204). The effects of continued Fludase treatment with concurrent *S. pneumoniae* colonization/infection are not fully elucidated.

As detailed above, late influenza infection leads to significant TRAIL-mediated apoptosis contributing to continued pathogenesis even as the viral load subsides. Pre-clinical data show that IAV-infected mice treated with anti-TRAIL sera had attenuated lung epithelial apoptosis, lung leakage and increased survival after IAV infection (155). Moreover, anti-TRAIL treatment was able to reduce bacterial load and protect against *S. pneumoniae* coinfection (205). Alternatively, Bcl-2 inhibitors which were developed to treat certain cancers are anti-apoptotic and have been suggested as potential treatment for influenza. *In vitro* data showed decreased viral replication and spread due to these agents (206, 207). Maintenance of the epithelial barrier and induction of antiviral mechanisms involve IFN signaling during influenza. Interferon-lambda treatment in mice leads to reduced viral load and improved survival without inducing a pro-inflammatory cytokine release (208). In another study, IFNλ treatment was able to prevent viral spread from the nasal passages to the lungs and confer resistance to IAV in mice for up to 6 days (209). However, in a model of IAV and methicillin resistant *Staphylococcus aureus*/Streptococcal superinfection, increased INFλ in IAV-infected mice lead to increased bacterial burden due to decreased bacterial uptake by neutrophils (210). It remains to be seen if any of these potential therapies will prove beneficial in treating human influenza.

Corticosteroids are routinely used for their anti-inflammatory properties in chronic conditions such as asthma and chronic obstructive pulmonary disease (COPD). Because influenza and ARDS manifests with a severe pro-inflammatory response, appropriately blunting that response may be beneficial during clinical illness. Additionally, corticosteroids have direct effects on the respiratory epithelium that may be protective. *In vitro* steroid treatment led to decreased epithelial permeability through the action of claudin-8 and occludin recruitment to TJs (211). However, corticosteroids were not found to be of benefit to patients during IAV infections (212, 213). A Cochrane review and another meta-analysis highlighted significant heterogeneity in published studies and did not show benefit but instead had a trend toward increased mortality (214, 215), and therefore, their efficacy as a therapy during influenza remains controversial.

*S. pneumoniae* is typically susceptible to many commonly used β-lactam antibiotics like penicillin. However, their resistance to multiple antibiotic classes is growing (216). Current vaccines for pneumococcal disease include 13-valent pneumococcal conjugate and 23-valent polysaccharide vaccines (217). Despite broad immunization practices however, invasive pneumococcal disease remains common with high morbidity and mortality. Similar to influenza, targeting the microbe-host interaction could provide novel treatment strategies for pneumococcal disease. One example is *S*-carboxymethylcysteine (S-CMC) which is a mucolytic agent used in COPD which has been shown to inhibit adherence to both pharyngeal and alveolar epithelia (218, 219).

## Conclusion

As a mucosal organ system with a large surface area and unremitting exposure to the external environment, protection of the respiratory barrier is of utmost importance to human health. Since barrier breach is a necessary first step for environmental pathogens to gain a foothold in the RT, maintaining the integrity of the mucosal barrier is a focus point of host defense and redundant mechanisms/pathways may be utilized to ensure its subsistence. Herein, we reviewed findings that pertain to crosstalk between structural cells and local leukocytes that play a role in immune defenses against IAV and *S. pneumoniae*. Although not covered here, the endogenous microbiome is likely to play an important role as a mediator of pulmonary immune responses during infection. The crosstalk at the interface of microbial pathogens and human host epithelium presents multiple opportunities for the development of clinically relevant therapies. Targeting host mechanisms may provide less opportunities for the emergence of pathogen resistance, and if used in combination with direct antimicrobial medications may prove superior to monotherapy.

As these pathogens evolve, it is imperative that additional information is garnered on interactions that occur between host cells and these agents as well as cell-cell crosstalk in order to discover more effective therapeutic strategies to overcome infection when the mucosal barrier is breached. It is also of importance to determine how these primary mechanisms relate to an individual with underlying chronic lung disease such as asthma, COPD, and interstitial pulmonary fibrosis, as the immune and structural architecture as well as the microbiome of these hosts are fundamentally different which likely leads to alterations in the defense mechanisms during respiratory infections.

## Author Contributions

All authors participated in writing and editing the paper and approved the final submission. Figures were drawn by AS (Figure 1) and MT (Figures 2, 3).

### Conflict of Interest

The authors declare that the research was conducted in the absence of any commercial or financial relationships that could be construed as a potential conflict of interest.
